# The management of space-occupying cyst in the tumor bed after brain tumor surgery: a case series integrated with literature synthesis

**DOI:** 10.1186/s41016-025-00409-3

**Published:** 2025-10-06

**Authors:** Zhiqiang Liu, Bei Liu, Chaoqun Weng, Zhixiong Lin

**Affiliations:** 1 Department of Neurosurgery, Sanbo Brain Hospital, Capital Medical University, Beijing, China; 2 Department of Medicine, Fujian Health College, Fuzhou, Fujian Province 350100 China; 3 Department of Neurosurgery, Fujian Sanbo Funeng Brain Hospital, Fuzhou, Fujian China

**Keywords:** Brain tumor, Postoperative, Complication, Space-occupying cyst

## Abstract

**Background:**

The postoperative space-occupying cyst (SOC) in the Tumor bed is rarely reported, and they are easily overlooked in the early stages. This oversight may result in serious consequences. This study aimed to systematically analyze the clinical characteristics and principles of managing SOC.

**Method:**

We conducted a retrospective analysis of clinical data on postoperative Tumor bed SOC at our institute. Comprehensive searches of English literature were performed on PubMed and Web of Science databases, while Chinese literature searches were conducted on the China National Knowledge Infrastructure and Wanfang Database, with a cutoff date of August 2024. Results Among 1026 brain Tumor resections performed at our institute, 10 patients (0.97%) had tumors situated in the supratentorial area. Four (40%) patients were managed with external drainage using an Ommaya reservoir placed in the cystic cavity, while six (60%) underwent direct percutaneous puncture drainage. A favorable prognosis was observed in all treated cases. A total of 106 cases were documented in both Chinese and English literature, yielding an incidence rate ranging from 0.04% to 4%. Percutaneous puncture external drainage was the predominant intervention, performed in 47 cases, representing the highest percentage at 44.3%. A favorable prognosis was observed in 78.1% (82/105) of treated cases, with four reported deaths.

**Conclusions:**

Supratentorial brain tumors situated within the cerebrospinal fluid circulation may give rise to SOC after resection. Following aggressive treatment, most patients experience a favorable prognosis.

## Background

The diagnosis and management of complications arising from cancer treatment constitute an evolving field [1]. Surgical resection remains a cornerstone in effectively treating common brain tumors. However, even with the improvements in perioperative care, the incidence and mortality following brain tumor resection remain high [2]. Most studies define the short-term postoperative period as 30 days for assessing outcomes and complications [2]. Generally, short-term postoperative complications can be broadly categorized into neurological, regional, and systemic events [3]. Such complications significantly increase the risks of mortality and neurological decline, often necessitating prolonged hospitalization, reoperation, readmission, and delayed initiation of adjuvant chemotherapy and radiotherapy [4].

Braunsdorf et al. [5] first documented space-occupying cystic lesions following malignant glioma resection, noting fulminant presentations including impaired consciousness, hemiparesis, aphasia, elevated intracranial pressure signs, and midbrain syndrome in two cases. Korinth et al. [6] similarly reported such lesions post-resection of diverse brain tumors (71% malignant). Notably, both cohorts included radiotherapy-exposed patients. Subsequent case reports predominantly describe space-occupying cystic lesions within 30 postoperative days in radiotherapy-naïve patients with benign/malignant tumors [7–10], paralleling our institution’s experience with 10 early-onset cases. This cyst, akin to focal hydrocephalus, manifests as a local space-occupying mass effect, resulting in intracranial hypertension, cerebral edema, local neurological dysfunction, and potentially life-threatening outcomes [11], necessitating heightened clinical awareness. Current nomenclature variations include “space-occupying cyst” [5], “cyst” [12], “expanding cyst” [13], “space-occupying cystic lesions” [6], “expanding cerebrospinal fluid cyst” [8], “symptomatic fluid collection” [14], and “space-occupying tumor bed cysts” [15]. Based on our cohort’s characteristics, we propose the standardized term “space-occupying cyst (SOC),” defined as follows: *an expansive space-occupying fluid collection emerging within 30 days post-resection in the tumor cavity that demonstrates serial radiographic enlargement with surrounding structural compression*. Current evidence remains limited to case reports, lacking comprehensive analyses of SOC’s clinical profile and therapeutic standards after intracranial tumor resection.

In this study, we conducted an in-depth analysis of the clinical features and management principles derived from the examination of the ten confirmed cases at our institute. Additionally, we performed a literature review of the relevant literature to explore the potential characteristics and treatment strategies associated with this lesion.

## Methods

Following approval from the institutional review board, a retrospective analysis was conducted on 1026 cases of surgically treated brain Tumors at our institute, spanning from January 2016 to January 2024.

### Inclusion criteria

1. Patients diagnosed with intracranial tumors requiring surgical intervention, confirmed by the following:
  ◦ Preoperative neuroimaging (MRI/CT)
  ◦ Postoperative pathological diagnosis via craniotomy
2. All patients underwent either of the following:
  ◦ Gross total resection (Simpson Grades I–II)
  ◦ Near-total resection (> 90% tumor volume reduction)
3. Postoperative status within 2–6 h of the following:
  ◦ Regained consciousness (GCS score ≥ 13)
  ◦ Postoperative non-contrast CT within 6–24 h showing:
  No hematoma in the tumor cavity
  No significant mass effect
4. New-onset neurological deterioration within 30 days postoperatively defined as follows:
  ◦ GCS score decrease to ≤ 12
  ◦ OR typical intracranial hypertension symptoms are as follows:
  Severe headache with vomiting
  Visual disturbances
  Altered mental status
5. CT confirmation upon neurological deterioration demonstrating the following:
  ◦ Space-occupying lesion in original tumor bed are as follows:
  Iso- to slightly hyperdense (compared to CSF)
  With surrounding tissue compression
  No significant enhancement
6. Refractory to conventional medical therapy are as follows:
  ◦ No improvement or only transient relief after the following:
    Mannitol (20%, 1–1.5 g/kg)
    Dexamethasone (10 mg IV q6h)
    For at least 48 h

### Exclusion criteria

1. Use of chemotherapeutic wafers (e.g., BCNU) in resection cavity
2. History of cranial radiation therapy within 1 year preoperatively or prior to symptom onset
3. Concurrent complications that may cause intracranial hypertension are as foolows:
  Intracranial hemorrhage
  Cerebral infarction
  Hydrocephalus
  Intracranial infection
  Tumor recurrence

Ethical approval was granted by the Ethics Committee of Fujian Sanbo Funeng Brain Hospital (No. FJSBNK-YJ-2024-002-01). This study was performed in accordance with relevant guidelines and regulations. The Declaration of Helsinki, as well as any later modifications or equivalent ethical standards, were followed by the current study. All included patients provided signed informed consent to participate in the study. This analysis focused on cases where patients developed local fluid accumulation near the surgical cavity, progressing to a SOC. Data on patient characteristics, tumor pathology, clinical symptoms, cyst location, size, imaging manifestations, disease course, treatment, and prognosis were collected.

The following keywords were used in PubMed and Web of Science for searching English literature: “space-occupying,” “cysts,” “cyst,” “cystic lesions,” “fluid collection,” “brain tumor*,” “glioma,” and “postoperat*.” Simultaneously, which were employed for the Chinese literature searches on China National Knowledge Infrastructure (CNKI) and Wanfang Database. The search was conducted until August 2024 in both English and Chinese databases. Two independent evaluators screened the abstracts of the relevant literature and subsequently performed a full-text review and data extraction of the identified studies.

## Results

### Patient characteristics and disease course

This case series comprised ten patients, including two (20%) males and eight (80%) females, with ages ranging from 30 to 65 years and an average age of 50.2 ± 11.09 years. Clinical symptoms and diagnostic imaging features indicated the emergence of SOC in the surgical cavity within 3–9 days post-surgery, averaging 5.3 ± 1.88 days (Table 1).

**Table 1:** Patients’ general clinical characteristics and treatment plan

Item		Numbers/proportions(%)
Gender		
	Male	2 (20%)
	Female	8 (80%)
Age (years)		50.2 ± 11.09
The time to find the tension cyst after surgery (days)		5.3 ± 1.88
Clinical symptoms		
	Disturbance of consciousness	10 (100%)
	Headache	4 (40%)
Pathology of neoplasia		
	Meningioma	1 (10%)
	Anaplastic oligodendrocyte tumour	4 (40%)
	Glioblastoma	5 (50%)
Primary or recurrent tumor		
	Primary	6 (60%)
	Recurrent	4 (40%)
Site of tumor lesion		
	Bilateral frontal concomitant corpus callosum	4 (40%)
	Right frontal concomitant corpus callosum	3 (30%)
	Left frontal concomitant corpus callosum	1 (10%)
	Right temporal lobe	1 (10%)
	Left sphenoid ridge	1 (10%)
Treatment		
	Puncture and drainage through bone window	6 (60%)
	Ommaya capsule drainage	4 (40%)

### Clinical symptoms and imaging features

**Fig. 1:**
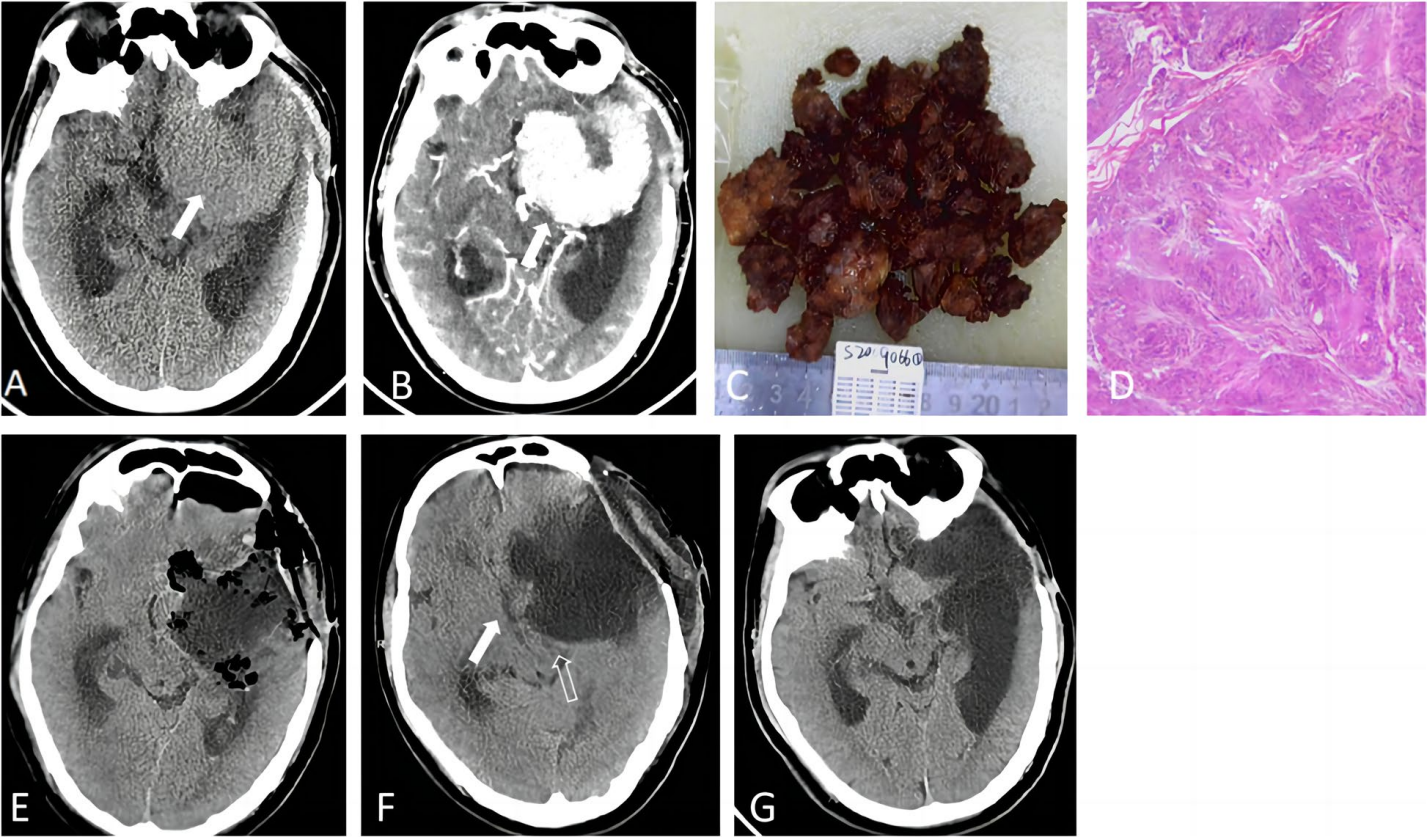
Imaging characteristics of the evolution of space-occupying cyst in the Tumor bed after reoperation for a huge recurrent Mengioma in the left sphenoid ridge of a 53-year-old male patient. **A** Brain computed tomography (CT) scan showed a sizable isodense spherical solid space-occupying lesion in the left middle cranial fossa (solid arrow), with its base attached to the left sphenoid ridge. The left mid-brain was compressed, and the temporal horns of the bilateral lateral ventricles enlarged. **B** Enhanced brain CT scan showed strong enhancement of the solid tumor (solid arrow). **C** Surgically excised tumor tissue. **D** HE staining showed meningothelial meningioma (WHO I). **E** Brain CT scan on the first day postoperatively exhibited a small amount of localized accumulation of fluid and air in the surgical cavity. **F** Brain CT scan on the fifth day postoperatively revealed an increase of localized accumulation of fluid in the surgical cavity, forming a space-occupying cyst (6.6 cm × 6.4 cm × 4.7 cm) that compressed adjacent brain structures (hollow arrow) and caused 1.1 cm rightward midline shift (solid arrow). **G** After percutaneous puncture for external drainage, the space-occupying cyst reduced in size. A follow-up brain CT scan at 1 year after surgery showed a small amount of fluid in the cystic cavity, without any space-occupying mass effect

**Fig. 2:**
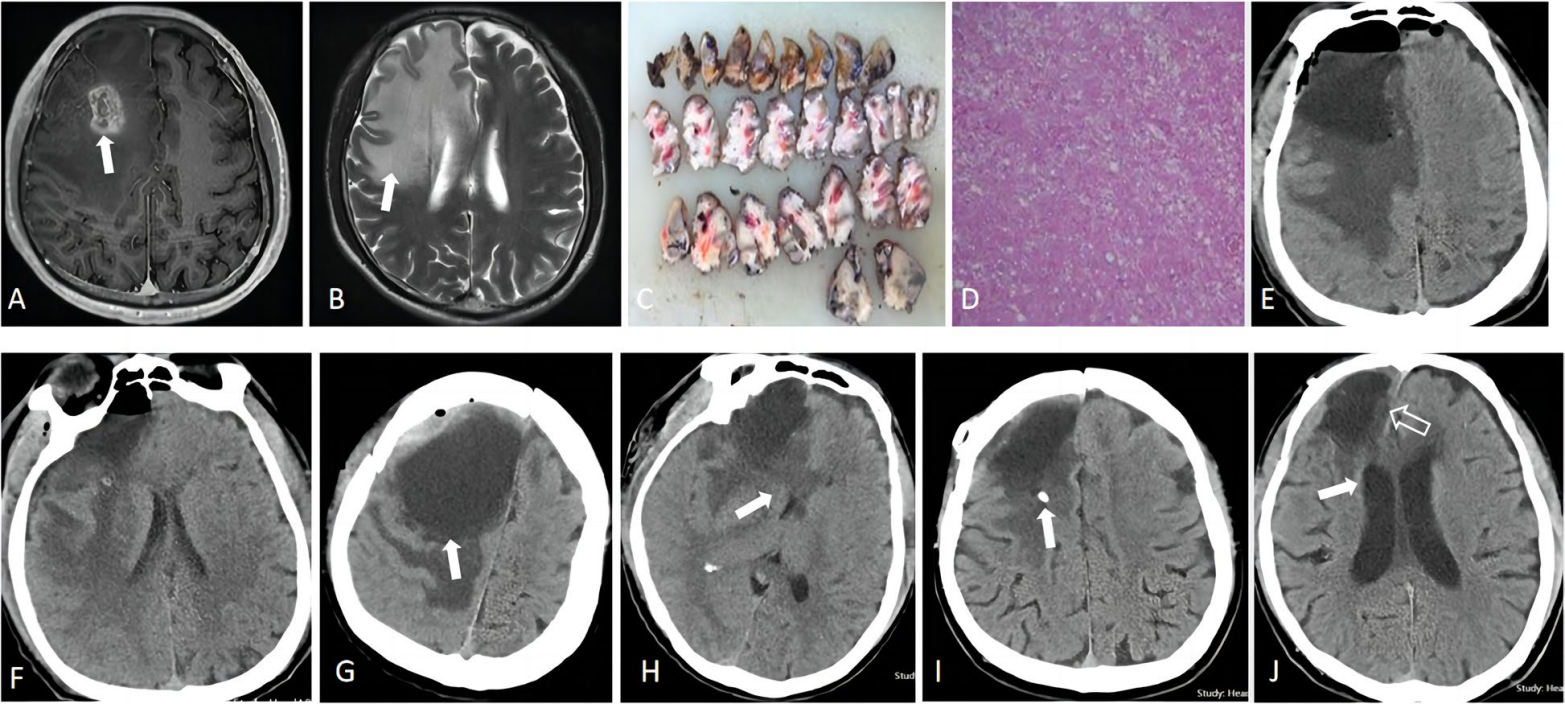
Imaging characteristics of the evolution of space-occupying cyst in the Tumor bed after reoperation for recurrent anaplastic astrocytoma in the right frontal lobe of a 45-year-old male patient. **A** Preoperative brain magnetic resonance imaging (MRI) T1-weighted images depicted a space-occupying hyperintense lesion in the right frontal lobe (solid arrow), accompanied by significant peritumoral edema. **B** Preoperative brain MRI T2-weighted image showed significant peritumoral edema with long T2 signal(solid arrow), the right frontal horn of the ventricle was compressed. **C** Surgically excised tumor tissue. **D** Postoperative HE staining suggested glioblastoma (WHO IV). **E**, **F** Subsequent brain CT scan on the first day postoperatively showed localized accumulation of fluid and gas in the surgical cavity and localized peritumoral edema. **G** Brain CT images on ninth day postoperatively revealed an increased accumulation of fluid in the surgical cavity had increased, forming a space-occupying cyst(6.0 cm × 7.1 cm × 7.0 cm), manifesting as a spherical shape (solid arrow), exerting compressed adjacent brain structures. **H** The ventricles showed obvious compression, and the frontal horn of the right lateral ventricle was largely absent (solid arrow), accompanied by a 1.0 cm midline shift to the left. **I** After external drainage through the reservoir placement, the cyst was reduced in size and space-occupying effect. A follow-up brain CT scan conducted 2 months after surgery showed only a small amount of localized fluid in the surgical cavity, peritumoral edema was significantly relieved, without any space-occupying mass effect (solid arrow). **J** The volume of fluid in the original cavity was significantly reduced (solid arrow), without any space-occupying mass effect, and the shape of the right ventricle recovered, without compression (hollow arrow)

All ten patients exhibited consciousness disturbance, characterized by lethargy that progressively worsened within one day. In severe instances, coma ensued, and headache was reported in four patients. Computed tomography (CT) imaging revealed a “spherical” enlarged low-density lesion within the surgical cavity following brain tumor resection. The surrounding tissues exhibited compression and swelling, resulting in the shrinkage or disappearance of the cerebral sulci (Figs. 1 and 2).

### Tumor pathology and location

Among the ten tumors, one was diagnosed as meningioma, and the remaining nine were high-grade gliomas. Four (40%) tumors were recurrent, while six (60%) were primary. In terms of tumor location, four (40%) tumors were located in the bilateral frontal lobes and corpus callosum, three (30%) in the right frontal lobe and corpus callosum, one (10%) in the left frontal lobe and corpus callosum, and one in the right temporal lobe. All nine (90%) patients with these tumor locations experienced intraoperative lateral ventricular opening. Additionally, one patient presented with an extracranial tumor at the left sphenoid ridge, causing severe compression of the contralateral fissure. In all patients, hemostasis during brain tumor resection was achieved using gelatin sponge and hemostatic gauze as hemostatic materials.

### Treatment

In four (40%) patients, an Ommaya reservoir was placed for external drainage, while percutaneous puncture drainage was performed in six (60%) patients. All patients received mannitol and furosemide to lower intracranial pressure and control cerebral edema. Following 1 week of external drainage of cystic fluid, the cystic cavities demonstrated reduction and stabilization. No recurrence was observed after the cessation and removal of drainage, and the medication for dehydration was gradually discontinued.

### Prognosis

After timely and aggressive treatment, all cysts demonstrated reduction and stabilization and all patients exhibited subsided cerebral edema. No new neurological dysfunction deficits were observed, and patients’ consciousness status returned to wakefulness. At the 3-month follow-up, the tumor bed and cavity remained stable and did not increase in size. Favorable prognosis was strictly defined as follows: (a) GOS ≥ 4 with cyst-related symptom resolution, and (b) imaging-confirmed space-occupying remission (stable/reduced cyst volume, midline shift < 3 mm, edema clearance) without recurrence. All cases met these objective criteria, demonstrating durable therapeutic efficacy.

## Literature review

A search of the literatures in PubMed and Web of Science identified nine eligible English studies (Table 2) [5, 6, 8–10, 14–17]. The reported incidence across the studies ranged from 0.04% to 4%, with a total of 82 cases. High-grade gliomas accounted for the majority, comprising 57.3% (47/82), followed by low-grade gliomas at 24.3% (20/82). Supratentorial Tumors represented 98.7% (81/82) of the cases. Among the 23 cases demonstrating an evident relationship between the Tumor and the lateral ventricle, 16 (69.5%) cases experienced intraoperative lateral ventricular opening. Percutaneous puncture for external drainage was performed in 33 cases, resulting in favorable outcomes in 75.7% (25/33) of cases. Other treatments included transcranial cystectomy, cyst fenestration and endoscopic cyst fenestration, cystoperitoneal shunt, cysto-atrial shunt, ventriculoperitoneal shunt, and cystoventricular stent placement. Overall, 83.9% (68/81) of the cases demonstrated favorable outcomes after treatment, while three cases expired with no reported cause of death.

**Table 2:** Review of English literature performed on PubMed and Web of Science databases on tumor bed cysts after brain tumor resections

Author (year)	Overall cohort (*N*)	Occurence of cyst (%)	Gender	Age	Histology	Location of the lesion	Interval after last tumor resection	Clinical presentation	Relationship between ventricular system and cerebrospinal fluid circulation pathways	Size of the cyst	Characteristics of drainage fluid	Therapy	Outcome
Braunsdorf W E et al. (1989) [5]	28	NR	12 F/16 M	51 YR	16 Cases of LGG, 7 cases of HGG, 2 cases of teratoma, 2 cases of malignant Meningioma, 1 case of metastasis	6 Cases of frontal lobe, 5 cases of parietooccipital lobe, 4 cases of parietal lobe, 3 cases of temporal lobe, 3 cases of frontotemporal lobe, 2 cases of temporoparietal lobe, 2 cases of frontoparietal lobe, 2 cases of third ventricle, 1 case of posterior fossa	8 WK	12 Cases of altered consciousness, 13 cases of hemiplegia, 4 cases of epilepsy, 3 cases of aphasia, 2 cases of coma, 6 cases of headache and vomiting	NR	45 mL (14–120 mL)	NR	Percutaneous external drainage	20 Cases improved, 5 cases unchanged, 3 cases deceased
Korinth M C et al. (2001) [6]	1240	31 (2.5%)	20 F/11 M	47 YR(12–74 YR)	21 Cases of HGG, 1 case of melanoma metastasis, 4 cases of LGG, 3 cases of chordoma, 2 cases of meningioma	10 Cases of frontal lobe, 8 cases of parietooccipital lobe, 4 cases of temporal lobe, 4 cases of pituitary/suprasellar region, 3 cases of parietal lobe, 1 case of occipital lobe, 1 case of cerebellum	17 Cases within 6 Mth (average 5.6 WK), 14 cases beyond 6 Mth (average 3.6 YR)	Increased intracranial pressure, such as headache, nausea, vomiting, altered consciousness (42%), or more focal neurological deficits, such as hemiplegia, aphasia, or cranial nerve deficits (45%); 4 cases of epilepsy (13%)	NR	40.3 mL (10–70 mL)	23 Cases showed cystic characteristics, 7 cases were yellow, 6 cases were clear, 6 cases were yellow–brown, and 4 cases were bloody. The protein content of the fluid was measured in 16 cases with an average of 22.9 g/l, ranging from normal (cerebrospinal fluid protein content: 0.21–0.42 g/l) to high (53 g/l)	5 Cases underwent repeated percutaneous puncture/external drainage, 5 cases underwent cyst excision surgery, 10 cases underwent cyst excision surgery with insertion of a Rickham catheter, 3 cases underwent endoscopic cyst fenestration surgery with insertion of a Rickham catheter, 6 cases underwent cystoatrial shunting surgery, and 2 cases underwent cystoperitoneal shunting surgery	Recovered well
Jinlu Yu et al. (2010) [7]	4782	2 (0.04%)	F	44 YR, 60 YR	HGG/meningioma	Right frontotemporal lobe, left sphenoid ridge	3D/6D	Drowsiness, dilated pupils, loss of light reflex in the right eye, left-sided limb paralysis/drowsiness, dilated pupil in the left eye, right-sided limb paralysis	2 Cases of compression of the lateral ventricle, with one case communicating with the lateral ventricle	40 mL/30 mL	Clear non-yellow fluid/clear accumulation fluid	Craniotomy for valve structure excision + 1 case of fenestration of the lateral ventricle and subarachnoid space	Recovered well, cyst disappeared after 3 months/cyst disappeared 10 months postoperatively
Andrea Talacchi et al. (2011) [8]	2	NR	M	65 YR, 53 YR	Left frontal lobe lung cancer brain metastasis/recurrent HGG	Left frontal lobe/right parietooccipital lobe	6D/3D	Consciousness impairment/hemiplegia accompanied by coma	No apparent impact	NR	NR	Craniotomy with external drainage/craniotomy	Recovered well
Thomas Beez et al. (2017) [15]	282	12 (4%)	5F/7M	53 YR (31–81 YR)	HGG	7 Cases of frontal lobe, 3 cases of parietal lobe, 1 case of temporal lobe, 1 case of occipital lobe	19 D (2–83 D)	11 Cases of increased intracranial pressure, 6 cases of newly developed focal deficits, and 3 cases of pseudomeningocele	11 Cases of ventriculostomy	NR	NR	7 Cases of cystoperitoneal shunting and 5 cases of ventriculoperitoneal shunting	9 Cases improved, 3 cases showed radiological improvement
Thomas Beez et al. (2018) [17]	1	NR	F	57 YR	Recurrent HGG	Right frontal lobe	2 Mth	Headache, pseudomeningocele, ventricular dilation	Lateral ventriculostomy	NR	NR	Endoscopic cyst fenestration surgery + lateral ventriculostomy, cyst-peritoneal shunting	NR
Takeshi Fujimori et al. (2019) [9]	1	NR	M	59 YR	Atypical meningioma	Left frontotemporal region	3 D	Motor aphasia	Left lateral fissure	NR	Deep red fluid	Neuroendoscopic placement of drainage tube for drainage	Recovered well
Yeong Jin Kim et al. (2021) [14]	1	NR	F	58 YR	Fibrous meningioma	Right frontal motor area	6 WK	Left upper limb monoplegia and paresthesia	No apparent impact	NR	NR	Mannitol and corticosteroid conservative treatment	Recovered well
Simon Schieferdecker et al. (2022) [10]	4	NR	1F/3M	50 YR (44–57 YR)	HGG	Left parietooccipital/right temporoparietal/left frontal/left parietooccipital lobe	3 WK, 6 Mth, 7 Mth, 1 D	Increased intracranial pressure, progressive subdural cerebrospinal fluid accumulation in 1 case	3 Cases communicating with the lateral ventricle	NR	NR	Placement of synthetic vascular graft stent into the cyst and lateral ventricle	Recovered well
Lin Yang et al. (2024) [16]	817	15 (1.84%)	7F/8M	55.13 ± 15.86YR (15–78 YR)	330 Cases of LGG, 487 cases of HGG,		7.07 ± 4.42D	GCS score dropped	NR	61.22 ± 17.35	The appearance of cystic fluid was markedly cloudier than the CSF. White blood cells and protein concentration were significantly higher in cystic fluid than in CSF. On the other hand, the concentrations of chloride ions and glucose were significantly lower than those of CSF	Bedside ultrasound-assisted puncture and drainage	Recovered well

*NR* not reported, *M* male, *F* female, *YR* year, *WK* week, *Mth* month, *D* day, *HGG* high-grade glioma, *LGG* low-grade gliomas

After searching the CNKI and Wanfang Database, six eligible studies were identified (Table 3) [11, 18–22], collectively reporting 24 cases with an incidence rate ranging from 0.04% to 0.75%. High-grade gliomas (12/24) and low-grade gliomas (2/24) together accounted for the majority of postoperative cases, at 58.3% (14/24). All Tumors were located supratentorially. Among the 18 cases with a clear relationship between the Tumor and the lateral ventricle, 13 (72.2%) cases underwent intraoperative lateral ventricular opening. Fourteen cases underwent percutaneous puncture for external drainage, while other management strategies included transcranial cyst fenestration, resection of valvular structures, and decompression surgery. No shunt surgeries were performed. The prognosis was favorable in 58.3% (14/24) of the cases, with one reported case of death attributed to severe cerebral herniation.

**Table 3:** Review of Chinese literature conducted on China National Knowledge Infrastructure and Wanfang Data on cystic lesions in the residual cavity after brain tumor surgery

Author (year)	Number of cases	Cyst incidence rate (%)	Gender	Age	Histology	Lesion location	Time of onset post tumor resection	Clinical presentation	Relationship with ventricular system and cerebrospinal fluid circulation pathways	Cyst size	Drainage fluid characteristics	Treatment regimen	Prognosis
Yu Jinlu et al. (2010) [18] (published in Chinese)	4782	2 (0.04%)	F	44 YR, 60 YR	HGG/meningioma	Right frontotemporal lobe/left sphenoid ridge	3D/6D	Drowsiness, dilated pupils (pupil diameter 4.0 mm), loss of light reflex in the right eye, and left limb paralysis/drowsiness, dilated pupil in the left eye, and right limb paralysis	2 Cases of compression of the lateral ventricle, with one case communicating with the lateral ventricle	40 mL/30 mL	Clear non-yellow fluid/clear accumulation fluid	Craniotomy for valve structure excision + fenestration of the lateral ventricle and subarachnoid space in 1 case	Recovered well
Luo Shengzhu et al. (2019) [19] (published in Chinese)	2	NR	F/M	79 YR, 54 YR	Brain hemorrhage/LGG	Right temporoparietal lobe/left frontal, insular, and temporal lobes	3D/9D	Consciousness began to deteriorate, gradually worsening, intracranial pressure reached 33 mmHg/gradual deterioration of consciousness	1 Case communicating with the lateral ventricle, 1 case with no impact	NR	Observed dark gray slightly viscous fluid/gelatinous substance and yellow fluid	Craniotomy with fenestration	Recovered well
Men Xuezhong et al. (2020) [20] (published in Chinese)	1	NR	M	32 YR	HGG	Bilateral frontal lobes, genu of the corpus callosum, right pons	3D	Headache, dizziness, nausea, progressive worsening of consciousness	Communicating with the lateral ventricle	NR	NR	External drainage with drainage tube placed through the original bone hole	Recovered well
Deng Yuxuan, et al. (2021) [21] (published in Chinese)	1060	8 (0.75%)	6F/2M	45.6 ± 16.7 YR (23–71 YR)	7 HGG, 1 LGG	2 Cases of left frontal lobe, 2 cases of left temporal lobe, 1 case of left parietooccipital lobe, 1 case of right frontal lobe, 1 case of right temporal lobe, 1 case of right parietooccipital lobe	7.8D (3–11D)	5 cases with worsening headache and deteriorating consciousness, 2 cases progressed to coma and developed brain herniation, 1 case with seizures, 5 cases with hyponatremia, 4 cases with fever, and 2 cases with anemia	NR (2 cases are communicating with the lateral ventricle)	154.375 cm (50–261.95 cm^3^)	One case had high protein content on fluid examination	5 Cases underwent cyst puncture drainage, 1 case underwent craniotomy with bone flap decompression after puncture, 1 case underwent puncture and fluid aspiration, and 1 case was treated with dehydration therapy	Symptoms all improved. GOS prognosis score: Good recovery in 3 cases, mild disability in 3 cases, severe disability in 2 cases
Zhang Zhongding et al. (published in Chinese) (2021) [11]	1	NR	M	35 YR	HGG	Right frontal lobe, genu of the corpus callosum	1D	24 h postoperatively, the patient suddenly experienced a decrease in consciousness, presenting with coma and dilation of the right pupil	Communicating with the lateral ventricle	NR	Large amount of pale red fluid	Craniotomy for decompression + temporal muscle fascia and original dura mater tension suturing	Death occurred 3 days postoperatively
Zhang Licheng et al. (2022) [22] (published in Chinese)	3505 (1470 cases were pathologically malignant, and 2035 cases were pathologically benign)	10 (0.28%)	5F/5M	51 YR (34–69 YR)	5 Cases of medulloblastoma, 2 cases of HGG, 1 case of central nervous system neuroblastoma, 1 case of lung cancer brain metastasis, 1 case of brain abscess	3 Cases in the left frontal lobe, 2 cases in the left temporoparietl lobe, 2 cases in the right parietal lobe, 1 case in the right trigone of the lateral ventricle, 1 case in the left lateral ventricle, and 1 case in the right frontal lobe	22.6D (3–50D)	Worsening of consciousness in 5 cases, worsening of limb motor function in 3 cases, worsening of language impairment in 1 case, and headache in 1 case	7 Cases of intraoperative ventricular system opening	NR	Many are yellow–brown or pale yellow, generally Turbid, may contain fibrous network structures, with occasional clear fluid; biochemical analysis of some cystic fluid shows protein content significantly higher than normal cerebrospinal fluid; 1 case had an osmotic pressure value of 364 mOsm/kg	3 Cases of craniotomy for cyst fluid removal, 7 cases of burr hole drainage	6 Cases recovered well, 2 cases had mild disability, and 2 cases had severe disability

*NR* not reported, *M* male, *F* female, *YR* year, *WK* week, *Mth* month, *D* day, *HGG* high-grade glioma, *LGG* low-grade gliomas, *GOS* Glasgow Outcome Scale

## Discussion

After surgical resection of a brain tumor, the initial phase typically involves the replacement of the local surgical cavity by fluid. Subsequently, the tumor cavity gradually diminishes due to the recruitment of surrounding brain tissues. However, in a minority of patients, the fluid within the local surgical cavity not only fails to diminish but also increases to form the SOC, forming a space-occupying mass effect and giving rise to symptoms such as intracranial hypertension.

The reported incidence rate of SOC ranges from 0.04% to 4% in the literature, predominantly occurring after resection of high-grade gliomas [7, 15]. In our case series, the observed incidence rate was approximately 0.97%, with gliomas being the predominant contributor. Notably, SOC occurrence timing varies: previous studies reported delayed presentations (> 1 month postoperatively), often associated with adjunctive therapies such as radiotherapy or carmustine wafer implantation [6, 15]. Korinth et al. [6] documented SOC in 2.5% (31/1240) of brain Tumor resections, with malignant gliomas accounting for 21/22 malignancies. Radiotherapy history existed in 52% (16/31) of patients and patients had undergone Tumor resection for more than once at the same location in 45% (14/31). Cyst development occurred within 6 months in 55% (mean 5.6 weeks) vs. > 6 months in 45% (mean 3.6 years). Similarly, Beez et al. [15] noted 4% incidence (12/282) after high-grade glioma resection, with mean onset at 19 postoperative days. Among these, six patients had concurrent radiotherapy and carmustine wafer implantation in the tumor bed, while two patients received carmustine wafers alone. In contrast, our cohort demonstrated early-onset SOC (mean < 14 days post-resection), consistent with non-adjuvant therapy cases.

### Pathogenesis

The mechanism underlying SOC may be attributed to various factors. (1) Mechanical obstruction by valve-like structures or morphological entrapment in flask-shaped cavities: Some researchers have confirmed the presence of a “valve”-like structure within the SOC through re-operation [7, 17]. Alternatively, post-resection cavities with narrow openings (“small-mouth, large-cavity” configuration) may entrap CSF entering through traumatic arachnoid defects [22]. Subsequent accumulation of hemoglobin degradation products and inflammatory mediators establishes hyperosmolar microenvironments. This osmotic gradient drives progressive fluid influx, creating a self-perpetuating cycle that expands cysts under space-occupying effects. Collectively, these mechanisms permit cerebrospinal fluid ingress while preventing egress. (2) Osmotic gradient from protein-rich exudates: A sizable tumor with a substantial traumatic area generates a significant amount of postoperative exudates. The elevated protein content in these exudates can impede cerebrospinal fluid reabsorption [23], thereby exacerbating the associated symptoms. (3) Dysfunction of meningeal lymphatic drainage (MLD): Emerging evidence indicates that MLD serves as a crucial pathway for cerebrospinal fluid (CSF) clearance and macromolecule elimination [24]. Craniotomy procedures may disrupt meningeal lymphatic vessels (MLVs), reducing CSF drainage efficiency. This compromises clearance of surgery-induced inflammatory mediators through MLVs. Consequently, accumulated inflammatory factors within the resection cavity increase vascular permeability, enabling plasma protein extravasation. These proteins elevate cyst fluid osmolality, thereby promoting space-occupying cyst formation. (4) Thrombin-mediated fibrogenesis: Experimental models demonstrate that subarachnoid hemorrhage activates TGF-β1, inducing arachnoid fibrosis that obstructs CSF dynamics [25]. SOC formation may result from synergistic interactions of these mechanisms.

In this case series, nine out of the ten patients underwent intraoperative lateral ventricular opening, and the remaining one case experienced severe compression of the lateral fissure, both closely associated with cerebrospinal fluid circulation. In certain instances, this was concomitant with dilatation and hydrocephalus involving all ventricles. Consequently, the localized formation of SOC after brain tumor surgery can be considered a specific and confined type of hydrocephalus. Similar cases have been observed by other researchers who have classified postoperative space-occupying fluid accumulation in the tumor bed into three types: isolated cysts, cysts with localized cerebrospinal fluid disturbance, and cysts with generalized cerebrospinal fluid disturbance [15].

#### Diagnosis

The key point of diagnosis for SOC: Suspect SOC when new neurological deterioration (impaired consciousness, focal deficits, or elevated ICP) occurs within 30 postoperative days (typically ≤ 7 days). Urgent CT/MRI demonstrate CSF-isointense cystic lesions (hypodense on CT/T1 hypointense, T2 hyperintense on MRI) with mass effect and perilesional compression, which confirms diagnosis. It is imperative to differentiate this condition from Pseudomeningocele, post-radiation cysts, cysts in the tumor cavity resulting from the implantation of chemotherapy wafers, bacterial abscesses, and tumor recurrence. Pseudomeningocele refers to cerebrospinal fluid (CSF) leakage into the epidural space through dural defects, forming encapsulated collections. These extradural lesions contain near-physiological CSF with low protein content. Clinically, they manifest as fluctuant masses without mass effect and demonstrate indolent progression [26], allowing straightforward differentiation. Post-radiation cysts are characterized by the absence of edema surrounding the lesion, lack of enhancement, and the absence of central necrosis on imaging. Post-radiation cysts in brain Tumors typically manifest later than common radiation necrosis, with an average reported progression of approximately 7.61 years [27]. Cysts may also arise following stereotactic radiotherapy for cerebral arteriovenous malformations. In a systematic review, the estimated rate of cystic formation was 3%, with a mean time to formation of 6.5 years [28]. Roux et al. [29] reported that in 122 adults with newly diagnosed supratentorial high-grade gliomas who underwent carmustine wafers implantation in the surgical bed, 22 (18.0%) patients developed postoperative contrast-enhanced cysts in the surgical bed, comprising 16 surgical bed cysts and 6 bacterial abscesses. Magnetic resonance imaging images of postoperative bacterial abscesses typically exhibit increased linear contrast along the abscess cavity wall, high signal on diffusion-weighted imaging, restricted diffusion on apparent diffusion coefficient, and occasionally, residual air within the cavity. The contrast-enhanced, thin, and rounded edges of carmustine wafers sharply contrast with the nodular appearance of recurrent tumors. MR perfusion-weighted imaging and MR spectroscopy can be valuable in recognizing early recurrence [30, 31].

### Management strategies

The principle of treatment is to drain the fluid and alleviate the space-occupying mass effect. Two types of interventions are employed: temporary interventions and permanent surgery. Temporary interventions encompass both surgical and non-surgical approaches. Non-surgical treatment involves the aggressive control of cerebral edema and tissue swelling post-surgery, aiming to prevent the reduction or closure of the surgical opening induced by severe cerebral edema. Surgical treatment options encompass puncture for external drainage and drilling for the placement of a fluid reservoir or external drainage. In conjunction with our cases and relevant reports in other literature, the accumulation of fluid in the tumor bed can be reduced or even absorbed through external drainage over time. This suggests that inflammatory exudates and damaged tissue cells from the traumatic area can be eliminated through drainage of the cystic cavity. Simultaneously, with the repair of inflamed tissue, the phenomenon of the unidirectional valve gradually dissipates, thereby restoring the circulation of abnormally accumulated local cerebrospinal fluid.

Permanent surgical options encompass ventriculoperitoneal shunt, cystoperitoneal shunt, cystoventricular shunt, as well as endoscopic or transcranial enlargement of the dural opening and the communication of the tumor cavity with the surrounding subarachnoid space [10, 15]. Beez et al. [15] performed seven cyst shunts and five ventricular shunts, with 75% of treated patients experiencing clinical benefit. In contrast to the opinions of permanent shunt implantation and transcranial or endoscopic fenestration, our cases achieved resolution through external drainage by percutaneous puncture or placement of a fluid reservoir. Symptomatic improvement was observed in conjunction with medication to control cerebral edema, obviating the need for permanent shunt implantation and minimizing the risk of post-shunt complications.

#### Prognosis

With timely diagnosis and intervention, SOC generally demonstrate favorable outcomes. In our case series, prompt recognition and management resulted in no residual severe neurological deficits. However, undetected SOC may culminate in fatal outcomes, as documented in published reports.

## Limitations

Our study had several limitations. First, its retrospective case series design introduces potential selection bias, as cases were derived from a single tertiary care center. Although SOC exhibit low incidence, our cases size further constrains statistical power. The heterogeneity in diagnostic criteria and therapeutic protocols across the literature synthesis precluded direct comparative efficacy assessments. Additionally, cyst fluid analysis was not performed, limiting biomolecular characterization.

## Conclusion

SOC constitute a clinically significant yet uncommon complication of intracranial tumor resection. Despite their relatively low incidence, delayed intervention may lead to severe neurological sequelae, necessitating protocolized neurological assessments. Postoperative monitoring is imperative for early lesion identification, with non-contrast cranial CT serving as the primary diagnostic modality when indicated. Prognosis remains favorable with prompt therapeutic intervention. Postoperative monitoring is crucial for timely lesion detection, and if necessary, prompt brain CT can confirm the diagnosis. Prognosis is generally favorable with aggressive treatment.

## Data Availability

No datasets were generated or analysed during the current study.

## Abbreviations

SOC: Space-occupying cyst
CNKI: China National Knowledge Infrastructure
CT: Computed tomography
